# Stimuli-Responsive Microjets with Reconfigurable Shape**

**DOI:** 10.1002/anie.201308610

**Published:** 2014-01-30

**Authors:** Veronika Magdanz, Georgi Stoychev, Leonid Ionov, Samuel Sanchez, Oliver G Schmidt

**Affiliations:** Current affiliation: Max Planck Institute for Intelligent SystemsHeisenbergstrasse 3, 70569 Stuttgart (Germany); Leibniz Institute of Polymer Research DresdenHohe Strasse 6, 01069 Dresden (Germany); Institute for Integrative Nanosciences, Leibniz Institute for Solid State and Materials Research DresdenHelmholtz Strasse 20, 01069 Dresden (Germany); Material Systems for Nanoelectronics, Technische Universität ChemnitzReichenhainer Strasse 70, 09107 Chemnitz (Germany); Technische Universität Dresden, Fakultät Mathematik und Naturwissenschaften01062, Dresden (Germany)

**Keywords:** microjets, micromotors, nanomotors, responsive polymers, self-folding

## Abstract

Flexible thermoresponsive polymeric microjets are formed by the self-folding of polymeric layers containing a thin Pt film used as catalyst for self-propulsion in solutions containing hydrogen peroxide. The flexible microjets can reversibly fold and unfold in an accurate manner by applying changes in temperature to the solution in which they are immersed. This effect allows microjets to rapidly start and stop multiple times by controlling the radius of curvature of the microjet. This work opens many possibilities in the field of artificial nanodevices, for fundamental studies on self-propulsion at the microscale, and also for biorelated applications.

Biological micro-swimmers, such as bacteria or sperm cells, are generally composed of flexible or moving parts, which thrust the propulsion of the micro-organism. The movement of biological swimmers is often provided by oscillation of a flexible tail powered by energy-rich chemical reactions (for example, hydrolysis of ATP),[1] and some of them contain small nozzles where jet forces are generated. Furthermore, they are able to respond to temperature, chemical,[2] pH, light, and other gradients.

Artificial self-propelled micro-nano-motors are engineered by multiple methods leading to a rich variety of architectures, such as nanowires,[3] microtubes,[4] spherical particles,[5] helical structures,[6] and more.[7] Those autonomous micromotors offer great possibilities for research from fundamental mechanisms of motion[5a,e, 8] at the micro- and nanoscale to potential biomedical[3b, 9] and environmental applications.[10] In contrast to biological swimmers, artificial micromotors are commonly formed by rigid structures. Some exceptions are bi-segmented nanowires containing flexible joints formed of silver or gold that allows their magnetically induced motion.[11] A particular case of artificial motors are catalytic motors, where the motion is generated by asymmetry of chemical reactions occurring on the structure of the micromotor.[3c,f, 12] One of the most common reactions employed in the field of catalytic micromotors is the catalytic decomposition of hydrogen peroxide into oxygen and water that takes place when artificial micromotors are immersed in that fuel solution. Catalytic tubular microjets are bubble-propelled,[4a,b, 13] can be externally guided by small magnetic fields,[14] accelerated and also stopped by temperature[15] and light,[16] which affect the generation rate of oxygen bubbles, and biofunctionalized[17] for selective isolation of different analytes.[18]

Most of the reported artificial microjets are based on inorganic materials. Very recently, Wang et al. reported the electrochemical fabrication of catalytic microjets containing Pt material in their interior coated with soft polymeric material.[4d] These microjets are, however, unable to change their shape while swimming, as their tubular structure is composed of compact walls. In contrast to rigid inorganic materials, stimuli-responsive polymers enable great variability in shape, size, and materials composition,[19] at the same time providing flexibility to the folded tube structure, which is not possible by electrochemical deposition of still rigid microtubes.

Herein, we present the fabrication of flexible thermoresponsive polymer microjets. These self-propelled microjets can reversibly fold and unfold in an accurate manner by applying changes in temperature to the solution where they are immersed. This effect allows them to start and stop multiple times by controlling the radius of curvature of the microtube. This property, added to its semi-transparency, makes this type of microjet especially attractive for studying fundamental questions such as the effect of curvature on the bubble nucleation and its correlation with the speed of microjets.

The self-folded polymer films are bilayers in which the active component is thermoresponsive poly(N-isopropylacrylamide) (PNIPAM), which swells below 32 °C and shrinks above this temperature. As a result, the PNIPAM-based bilayers fold and unfold at reduced and elevated temperature, respectively.[20] The folding/unfolding is fully reversible and can be repeated many times. Moreover the shape of the folded object can be precisely controlled by the 2D shape of the films in the way that tubes, scrolls, rounded capsules, pyramids, and other complex three-dimensional structures are possible.[20d, 21]

We took advantage of the reversible folding/unfolding of polymer films for the design of self-propelling microjets, the movement of which can be temporarily controlled by an external stimulus, namely temperature. In a typical experiment, a photo-cross-linkable derivative of PNIPAM (poly(NIPAM-BA)), which swells and shrinks in water below and above 28 °C respectively, is dip-coated from its ethanol solution on a silicon wafer. Afterwards, polycaprolactone (PCL) with 2 wt % of benzophenone is dip-coated from toluene solution on the poly-(NIPAM-BA) film. The thickness of poly(NIPAM-BA) and PCL films are 2 μm and 600 nm, respectively. The bilayer film is illuminated through a photomask for 75 min by a halogen lamp with intensity of 150 μW cm^−2^ to cross-link the polymers. The illuminated film is then rinsed in chloroform to remove the polymers from the non-irradiated areas. The photopatterned polymer bilayer structures on the silicon wafer are coated with 0.5 nm of platinum using a Leica EM SCD 500 sputter coater, equipped with a Leica EM QSG 100 control unit.

During the temperature experiments, the silicon wafer with the polymer films is immersed in pure water to induce the swelling of the thermoresponsive layer at room temperature. The polymer-Pt-trilayer remains undeformed in aqueous media above 28 °C (Figure 1 a,c). Cooling leads to swelling of the thermoresponsive polymer, bending of the films, and eventually to the formation of micrometer-sized tubes with diameters of about 30 μm (Figure 1 b,d). The thermoresponsive polymers react almost instantaneously to the temperature changes, so the rolling–unrolling process occurs rapidly, as can be observed in the real-time videos (see the Supporting Information). It was found that the thin Pt layer has almost no effect on the diameter of the tubes. Reversible heating leads to the complete unfolding of the tubes. Thus, the rolling and unrolling is completely reversible and can be repeated many times. After the first folding of polymer films is observed, the sample is transferred to a hydrogen peroxide solution containing 1–5 wt % H_2_O_2_ and 1 % Triton X as surfactant. An AxioScope microscope equipped with a high-speed camera is used to record videos at 60 frames per second. A Peltier element and thermocouple are used to heat and cool the sample and monitor the temperature.

**Figure 1:**
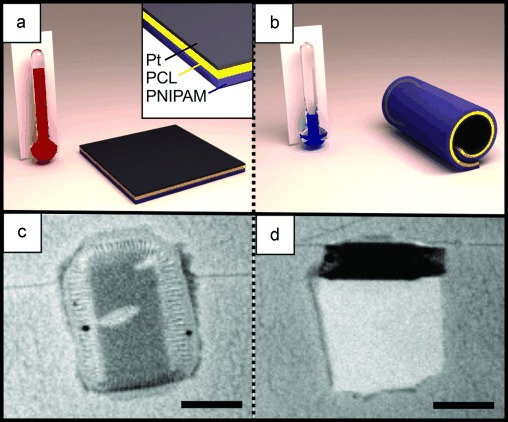
Representation (a, b) and microscopy images (c, d) of reversible rolling–unrolling of polymer-Pt films. The inset in (a) shows the 3 layers of poly(NIPAM-BA), PCL (polycaprolactone), and platinum. Scale bars: 50 μm.

Once immersed in H_2_O_2_ solution, we investigated the formation of oxygen bubbles both on flat films and in tubular jets. We did not observe release of bubbles from flat films owing to quick diffusion of the formed oxygen from the platinum surface. In this case, the diffusion rate of oxygen is apparently faster than its generation rate and the local oxygen gas concentration does not approach the solubility limit (Figure 2 a, *t*=0 s to *t*=2.6 s). The formation of bubbles was observed only when the film was rolled, allowing the generated oxygen molecules to accumulate into visible bubbles[4a] (Figure 2 a, *t*=5 s to *t*=5.1 s and Figure 2 b, *t*=0 s to *t*=6 s)). In this case, oxygen formed inside the tube does not have enough time to diffuse out of the tubes, and its local concentration is high enough to form visible bubbles. When the film unfolds again, the formation of bubbles ceases (Figure 2 b, *t*=9 s to *t*=13 s).

**Figure 2:**
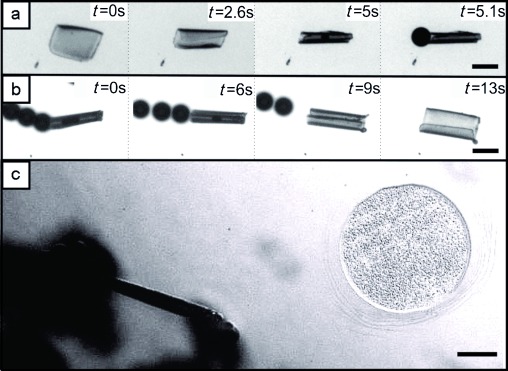
Dependence of formation of bubbles on the shape of the film; the visible oxygen bubbles are formed only in the cavity of a tube. a) Cooling of stimuli-responsive films below 28 °C and folding process over 5.1 s (see the Supporting Information, Video S2a). b) Unfolding by warming up with stopping of bubbles (see the Supporting Information, Video S2b). c) Comparison of a very actively bubbling folded tube (left) and unfolded non bubbling films (right) under the same fuel conditions (2 wt % H_2_O_2_ and 0.1 % Triton X) (see the Supporting Information, Video S2c). Scale bar: 100 μm in (a) and (b), 50 μm in (c).

To rule out other possible effects, a comparative study was carried out by recording videos of two micropatterned structures with exactly the same trilayers: one folded and another unfolded (Figure 2 c, left and right, respectively). The left polymer layer in Figure 2 c folded owing to the temperature decrease below 28 °C. The right polymer layer remained flat and did not respond to the temperature stimuli owing to some defects in the polymer film that occurred during the fabrication process. This defect can be caused by insufficient removal of non-illuminated polymer causing the polymer film not to release its edges from the substrate which is necessary for the folding mechanism. The samples were immersed in the same H_2_O_2_ solution (2 wt % and 0.1 % Triton X) under the same temperature conditions. The results confirm that under the same conditions, the hollow structure of the tube is indeed essential to generate the bubbles, whereas planar films do not generate any visible bubbles. The solubility of oxygen gas increases at lower temperatures, thus after roll-up, the generation of bubbles was even less likely to happen compared to the case of an unfolded tube at high temperatures. Thus, we conclude that the folded shape is essential for formation of the bubbles and eventually for the propulsion of tubular micromotors.

We used the dependence of the formation of bubbles on the shape of the polymer film (folded or unfolded) to control the movement of self-rolling microjets in H_2_O_2_ solutions. Figure 3 plots the speed and bubble frequency in dependence of the tube radius of a 265 μm long polymer jet. At low temperature (*T*<28 °C), we observe bubble release from the microjets that rapidly move (Figure 3 b, inset). Slow increase of the temperature leads to gradual unfolding of the films and an increase of its radius. As a result, the velocity reduces and the tube slows down (Figure 3 a). Upon increasing the temperature, the tube opens significantly (Figure 2), producing a lower frequency of bubbles that serve to propel the microjets (Figure 3 b). The opening of the tube maintains the catalytic properties over a certain range of tube radii so that it is still capable of generating large amounts of bubbles up to a tube radius of 30 μm, where the speed is maximum (Figure 3 a). Tubes that are larger than 70 μm in radius do not produce bubbles and consequently do not move. By controlling the temperature of the solution, we can tune the diameter and thus the speed of the microjets. We used incomplete unfolding of the tubes to quantitatively study the dynamic influence of the radius on the speed of microjets. Figure 4 shows that cycling of temperature above and below 28 °C can be employed to accelerate and slow down the polymeric Pt-jets. This speed control method is completely reversible and repeatable. The velocity of the conically shaped 265 μm long flexible polymer-Pt microjet can be as high as 300 μm s^−1^ in the completely folded state when its radius is about 30 μm. We note that large polymeric tubes sometimes sediment at the bottom surface of the petri dish and considerable friction impedes their maximum speed.

**Figure 3:**
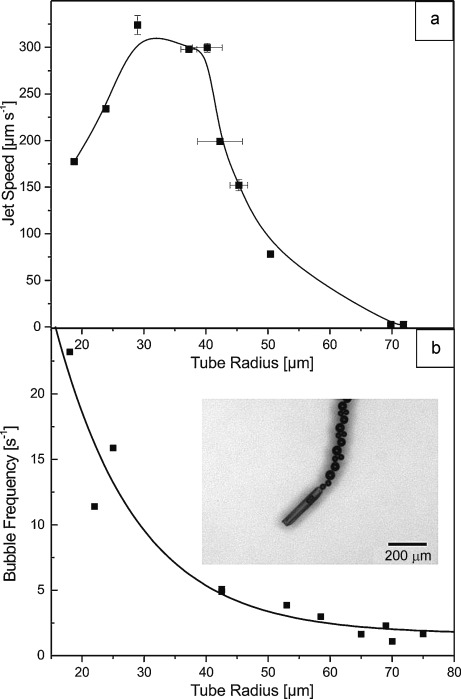
Dynamics of a flexible microjet with variable diameters. a) Microjet speed versus tube radius and b) bubble frequency versus tube radius. Inset: representative example of bubble-propelled polymeric jets. Error bars: standard deviation for *n*=3.

**Figure 4:**
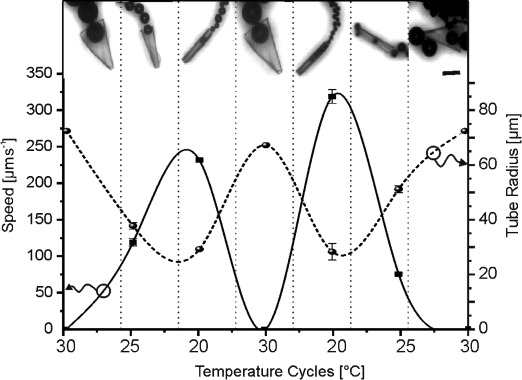
Cycles of folding and unfolding of thermoresponsive 265 μm-long polymer-Pt jets in 5 wt % H_2_O_2_ solution with 0.1 % Triton X as surfactant by cooling below and heating above 28 °C. The graph plots the average speed [μm s^−1^] (left *y*-axis, continuous line) and tube radius [μm] (right *y*-axis, dotted line) of polymer-Pt-microjets over the temperature cycles. Insets: snapshots of Pt-polymer jets with changing radius owing to the compressing at temperatures below 28 °C and expanding at temperatures above 28 °C. Scale bar: 100 μm (Supporting Information, Videos S4a and S4b). Error bars: standard deviation for *n*=3.

When the polymer-Pt microjet partially unfolds, it reduces its bubbling frequency in agreement with Figure 3 and slows down and eventually stops (see the transition from 20 to 30 °C in Figure 4). When the temperature is decreased again below 28 °C, the jet can self-propel again once the tube folding reaches a minimum radius of about 45 μm. A radius of above 70 μm means the tube is unfolded and displays an opened polymer film. We performed several cycles from 20 to 30 °C where microjets were completely stopped (three times at 30 °C) and acquired maximal speed at temperatures close to 20 °C when the radius of the tubes is minimal.

These results indicate that the radius of the tubular microjet and consequently the size of the released bubbles dramatically influence the speed of bubble-propelled microjets. Future research should be devoted to the optimization of the size and design of thermoresponsive polymeric microjets of different shapes and dimensions.

In conclusion, we have demonstrated that the movement of thermoresponsive polymeric Pt microjets can be dynamically controlled by temperature changes. The folding mechanism changes reversibly the shape of the flexible polymer Pt jets, which in turn influences the dynamics of the microjets when they are self-propelled in peroxide solutions. We also showed that curvature is a crucial parameter for the formation of bubbles on a Pt film in microjets.

The speed of microjets is dependent on the radius of the microtube; thus, jet propulsion can be controlled in situ by tuning the radius during the actuation process and not only by fabrication before operation of the jet, as known from previous studies. This work can pave the way for future studies on bubble formation in cavities, as the polymeric jets are transparent and bubble formation inside the tubes can be optically studied. Furthermore, the outer polymer film can easily be functionalized with proteins or other bioactive substances. This system will be useful for developing flexible, biodegradable micromotors for various applications. The speed control by shaping of the polymeric Pt films offers a convenient control mechanism during operation of this new kind of flexible bubble-propelled micromotor.

## Experimental Section

*N*-isopropylacrylamide (NIPAM, Aldrich), 4-hydroxybenzophenone (Fluka), polycaprolactone (*M*_n_=70 000–90 000, Aldrich), benzophenone (Aldrich), and acryloyl chloride (Fluka) were used as received.

Synthesis of 4-acryloylbenzophenone (BA): 4-Hydroxybenzophenone (20 g, 0.1009 mol), diisopropylethylamine (19.3 mL, 0.1110 mol), and dichloromethane (80 mL) were added into a 200 mL three-necked round-bottom flask fitted with an overhead stirrer, a thermometer, and an addition funnel with acroloyl chloride (9.02 mL, 0.1110 mol) solution in dichloromethane (20 mL). The acroloyl chloride solution was added dropwise into the flask under cooling (0–5 °C) for ca. 3 h. The methylene chloride was removed by rotary evaporation. The residue was washed with 20 % HCl (80 mL), a saturated solution of sodium hydrocarbonate (80 mL), and dried over sodium sulfate. The solution was passed through a silica gel column with chloroform as the eluent. Chloroform was removed by a rotary evaporator. Finally, 24.44 g (95 %) of BA was obtained.

Synthesis of poly(NIPAM-BA): BA (0.02253 g, 0.089 mmol; 0.04551 g, 0.18 mmol; 0.11737 g, 0.47 mmol), NIPAM (1 g, 0.0885 mol), and AIBN (0.01453 g, 0.089 mmol) were added in 10 mL test tubes. Components were dissolved in 1,4-dioxane (6 mL) and degassed with nitrogen for 30 min. Test tubes were tightly sealed and placed into a shaker (70 °C, 90 rpm) for 24 h. Then the poly(NIPAM-BA) polymerization mixtures were cooled to room temperature and poured slowly into diethyl ether. Products were filtered and dried under vacuum.

Synthesis of poly(MMA-BA): MMA (6.2803 g, 62.72 mmol), BA (0.2405 g, 0.96 mmol), and AIBN (0.052 g, 0.31 mmol) were dissolved in toluene (30 mL). The mixture was purged with nitrogen for 30 min. The polymerization was carried at 70 °C under nitrogen atmosphere with mechanical stirring overnight. After cooling, the mixture was poured in 750 mL diethyl ether, and the precipitate was filtered and dried in vacuum at 40 °C.

Preparation of polymer bilayers: Poly(NIPAM-BA) was spin-coated from its chloroform solution on silica wafers. Polycaprolactone with 2–5 wt % of benzophenone or poly(MMA-BA) was spin-coated from toluene solution on poly(NIPAM-BA) film. The bilayer film was illuminated through TEM grid by halogen lamp for 75 min to cross-link polymers. The illuminated film was rinsed in chloroform to remove polymers in non-irradiated areas.
