# Supplementary material for: Stimuli-Responsive Microjets with Reconfigurable Shape
Source: Angew Chem Int Ed Engl. 2014 Jan 30;53(10):2673–7. doi: 10.1002/anie.201308610 (PMC4255230; doi:10.1002/anie.201308610)
Supplement: Supplementary file 1 [file anie0053-2673-sd1.pdf]

Angewandte Chemie
Eine Zeitschrift der Gesellschaft Deutscher Chemiker

Supporting Information

© Wiley-VCH 2014

69451 Weinheim, Germany

Stimuli-Responsive Microjets with Reconfigurable Shape**

Veronika Magdanz, Georgi Stoychev, Leonid Ionov, Samuel Sanchez,* and Oliver. G. Schmidt*

anie_201308610_sm_miscellaneous_information.pdf

Supporting Information

5 Movies:

z308610_VideoS2c.avi
z308610_VideoS4a.avi
z308610_VideoS4b.avi
z308610_VideoS2a.avi
z308610_ViedoS2b.avi
